# The Effects of Aerobic-Resistance Training and Broccoli Supplementation on Plasma Dectin-1 and Insulin Resistance in Males with Type 2 Diabetes

**DOI:** 10.3390/nu13093144

**Published:** 2021-09-09

**Authors:** Ayoub Saeidi, Mohammad Soltani, Ali Daraei, Hanieh Nohbaradar, Marjan Mosalman Haghighi, Nikoo Khosravi, Kelly E. Johnson, Ismail Laher, Anthony C. Hackney, Trisha A. VanDusseldorp, Hassane Zouhal

**Affiliations:** 1 Department of Physical Education and Sport Sciences, University of Kurdistan, Sanandaj 66177-15175, Iran; saeidi_as68@yahoo.com; 2 Department of Biological Sciences in Sport, Faculty of Sports Sciences and Health, Shahid Beheshti University, Tehran 19839-69411, Iran; m.soltani904@gmail.com; 3 School of Health & Exercise Sciences, The University of British Columbia, Kelowna, BC V1V 1V7, Canada; alidaraei01994@gmail.com; 4 Faculty of Sports and Exercise Sciences, Alzahra University, Tehran 19938-93973, Iran; haniye70_sa@yahoo.com (H.N.); nikukh@alzahra.ac.ir (N.K.); 5 Faculty of Medicine and Health, The University of Sydney, Sydney 2006, Australia; marjan.mosalmanhaghighi@sydney.edu.au; 6 Department Kinesiology, Coastal Carolina University, Conway, SC 29526, USA; kjohns10@coastal.edu; 7 Department of Anesthesiology, Pharmacology & Therapeutics, The University of British Columbia, Vancouver, BC BV6T 1Z3, Canada; ismail.laher@ubc.ca; 8 Department of Exercise & Sport Science and Department of Nutrition, University of North Carolina, Chapel Hill, NC 27599, USA; ach@email.unc.edu; 9 Department of Exercise Science and Sport Management, Kennesaw State University, Kennesaw, GA 30144, USA; 10 M2S (Laboratoire Mouvement, Sport, Santé)-EA 1274, University of Rennes, F-35000 Rennes, France

**Keywords:** aerobic-strength training, concurrent exercise training, HOMA-IR, inflammation, body mass index, fat percent, broccoli

## Abstract

Background: This study aimed to evaluate the effects of a combination of aerobic-resistance training (CARET) and broccoli supplementation on dectin-1 levels and insulin resistance in men with type 2 diabetes mellitus (T2D). Methods: Forty-four males with T2D were randomly allocated to four groups (*n* = 11 each group): CARET + broccoli supplement (TS), CARET + placebo (TP), control + broccoli supplement (S), and control + placebo (CP). CARET was performed three days per week for 12 weeks. TS and S groups received 10 g of broccoli supplement per day for 12 weeks. All variables were assessed at baseline and 12 weeks. Results: Plasma dectin-1 levels were decreased in TS and TP groups compared with the CP group (*p* < 0.05). Cardiometabolic risk factors showed significant reductions in TP and TS groups compared to S and CP groups (*p* < 0.05). Conclusion: The combination of CARET and broccoli supplementation produced the largest improvements in insulin resistance and dectin-1 and other complications of T2D.

## 1. Introduction

Type 2 diabetes mellitus (T2D) is a global epidemic with high morbidity and mortality rates [1]. Risk factors such as obesity, unhealthy eating habits, and a sedentary lifestyle increase the risk of T2D [2,3]. Both obesity and T2D are associated with increased oxidative stress and inflammatory markers, which also have detrimental effects on the immune system [4].

Dectin-1 is a member of the C-type lectin receptor family that is highly expressed in macrophages and dendritic cells [5]. Dectin-1 is an innate immune receptor involved in various cellular responses, including in chronic inflammatory conditions such as autoimmunity and T2D [5,6]. Activation of dectin-1 induces the production of pro-inflammatory cytokines, chemokine [6], and reactive oxygen species [7], and dectin-1 is suggested to play a role in obesity-associated inflammation and insulin resistance, making it a therapeutic target in the treatment of chronic inflammation in insulin-resistant individuals [8].

Broccoli and broccoli sprouts are rich sources of bioactive compounds such as isothiocyanates, antioxidant vitamins, and selenium [9]. A key component of broccoli is sulforaphane, which reduces lipid peroxidation, increases total antioxidant capacity, and decreases oxidative stress by activating antioxidant pathways in T2D patients [10]. It has been reported that four weeks of supplementation with broccoli (10 g/d) decreases serum insulin concentrations and homeostatic model assessment of insulin resistance (HOMA-IR) in individuals with T2D [11]. Furthermore, sulforaphane (i.e., sulfur-rich compound found in cruciferous vegetables such as broccoli) inactivates NF-kB (nuclear factor kappa-light-chain-enhancer of activated B cells) inhibition of cytokine production, suggesting a possible beneficial effect in TD2 [11,12]. Administering sulforaphane increases muscle strength, improves muscle function and exercise capacity, and protects muscle from oxidative damage and inflammation [13]. Moreover, sulforaphane activates nuclear factor erythroid 2-related factor 2 (Nrf2) and reduces exercise-induced muscle fatigue by upregulating cellular antioxidant mechanisms and phase 2 enzymes involved in conjugation reactions [14]. Sulforaphane also decreases markers of muscle damage, such as creatinine phosphokinase and lactate dehydrogenase [15]. Although exercise can increase pro-inflammatory cytokines in the liver, kidney, and intestine [16,17], sulforaphane induces phase 2 enzymes through Nrf2 activation to reduce oxidative insults [16]. In addition, dietary supplementation with broccoli extract reduces exercise-induced muscle soreness and oxidative stress markers in T2D patients [18].

It is well known that changes in lifestyle such as a healthy diet and increased physical activity can attenuate the complications of T2D [19]. Combined exercise training aerobic + resistance (CARET) has recently been recommended for managing T2D; CARET may be more effective than either aerobic or resistance training alone in improving insulin resistance, inflammatory markers, glucose transporter type 4 (GLUT-4) expression (by increasing muscle mass), body weight, visceral fat mass, and lipid profiles in individuals with obesity and T2D [19,20]. In addition, CARET improves immune function by converting M1 macrophages to M2 subtypes, so reducing inflammation in obesity and T2D [21,22]. Although exercise training is often performed on most days of the week, CARET could have deleterious effects on inflammatory factors, endothelial function, and hemodynamic balance in sedentary individuals with obesity and T2D [22,23]. Therefore, CARET, which engages more groups of exercising muscles and requires greater energy expenditure, can increase pro-inflammatory factors, especially in the first month of the intensive exercise training program [24]. These findings emphasize the importance of tailoring optimal guidelines of aerobic and resistance training plus an ergogenic aid in individuals with T2D, especially in a population of sedentary individuals with obesity and T2D, whose pro-inflammatory factors are higher than their counterparts who have a healthy lifestyle [24].

Both CARET and broccoli supplementation separately improve obesity, diabetes, and inflammatory markers [11,22], although the effects of a combination of the two on plasma dectin-1, cardiometabolic risk factors, and body composition variables are unknown. Therefore, we hypothesized that 12 weeks of combined CARET and broccoli supplementation would have greater effects on cardiometabolic variables in individuals with T2D than the benefits from using either exercise training or broccoli supplement in isolation.

## 2. Materials and Methods

### 2.1. Participants

**Figure 1:**
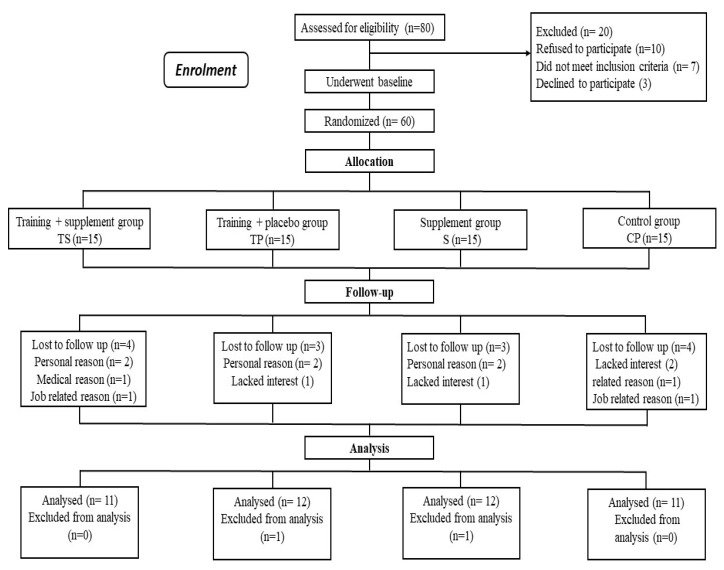
Participants flow chart. CP, Control–Placebo; S, Supplement; TP, Training–Placebo; TS, Training–Supplement.

Forty-four men (see Table 1 for participant characteristics) clinically diagnosed with T2D participated in this study. They were recruited from local hospitals and health centers through advertisements (i.e., poster/flyer, email, and social media applications). A sports medicine physician and an endocrinologist screened all participants to ensure they were healthy enough to participate in the study. Inclusion criteria included: patients with T2D as defined by the American Diabetes Association, the age range of 40 to 60 years, duration of diabetes >2 years, fasting plasma glucose concentration of 7.0 mmol/L or higher (i.e., 126 mg/dL or higher), and being treated with an oral hypoglycemic medication. Subjects were excluded if they had: hypertension (>160/90 mmHg) or other cardiovascular diseases, musculoskeletal disorders, respiratory diseases, neurological disorders, renal dysfunction, or other disorders that could limit physical activity (Figure 1). A written consent form was obtained from all subjects before the study. All subjects participated in a familiarization session where they were acquainted with the study procedures, benefits, and potential risks. All study procedures and protocols were approved by the local University Research and Ethics Committee (Ethics code: IR-IAU1397–3) and performed according to the latest revision of the Declaration of Helsinki [25].

### 2.2. Experimental Design

**Figure 2:**
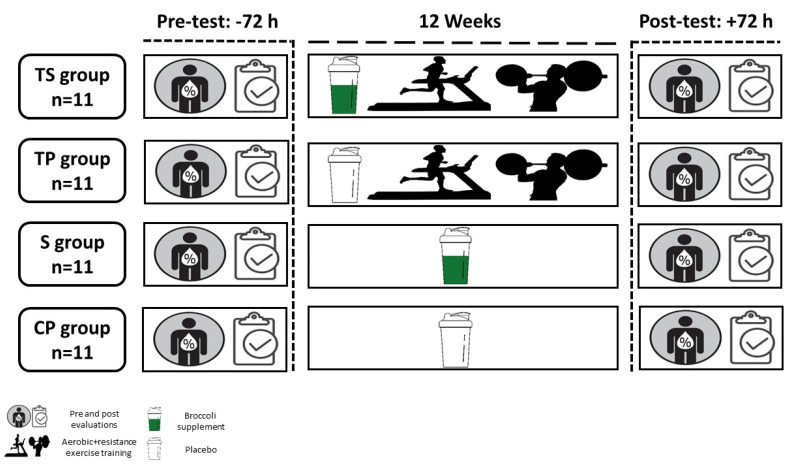
Experimental design. CP, Control–Placebo; S, Supplement; TP, Training–Placebo; TS, Training–Supplement.

Subjects were randomly assigned to four study groups (*n* = 11 per group): training + broccoli supplement (TS), training + placebo (TP), control + broccoli supplement (S), and control + placebo (CP) (Figure 1 and Figure 2). The randomization was conducted using a random number table. An independent coordinator (a dietitian) who was not involved in the study assigned participants to the TS, TP, S, and CP groups, and all participants were instructed to continue with their current medications and maintain their diets. Participants in the control group were instructed to maintain their current lifestyles until the end of the study. All participants were asked to fast for 12 h, avoid strenuous exercise for two days, and remain well hydrated prior to blood draws that were taken 48 h before the start of the program and 48 h after the 12-week exercise training program. Height and body mass were measured to determine BMI (weight (kg) divided by height^2^ (meters^2^)) before and after the study. Three familiarization sessions that included a 5- to 10-min warm-up, 20 min of resistance exercise, followed by performing 20 min of aerobic exercise at a moderate intensity, were held before starting the study.

### 2.3. Cardio-Respiratory Fitness, Body Composition and One-Repetition Maximum Assessments

A modified Bruce protocol using a motorized treadmill (Pulsar 3p, H/P/Cosmos, Nussdorf-Traunstein, Germany) with a gas analyzer system (Metalyzer 3B Analyzer, Cortex Biophysik, Leipzig, Germany) was used to estimate peak oxygen uptake (VO_2peak_) in a temperature-controlled room (21–23 °C). Blood pressure was recorded during the test using an automated blood pressure system (Omron M6 Comfort, HEM-7221-E, Omron Healthcare, Kyoto, Japan) and heart rates (HR) (beats/minute) were continuously recorded using an HR monitor (Polar Electro, Espoo, Finland). Participants reported their perceived exertion rating using the Borg scale (6–20) during the last 10 s of each phase of the test [26]. VO_2peak_ was determined by using the following criteria (According to American College of Sports Medicine guidelines for CPET test): (A) exhaustion based on a Borg scale of >17, (B) if the supervising physician observed or if the subjects reported dyspnea, dizziness or any contra indicatory symptoms, (C) a plateau in VO_2_ and (D) respiratory exchange ratio (RER) ≥1.10. Body fat percentage (%) and fat free mass (FFM) were measured by a bio-impedance analyzer (Medigate Company Inc., Dan-dong Gunpo, Korea) before and after the exercise training program. One-repetition maximum (1-RM) was determined during the last familiarization session. After a five minute warm-up, participants lifted the maximal amount of weight that could be lifted for 6–8 repetitions for each exercise (described below), and then 1 RM was calculated according to the Brzycki formula [27]. Participant rested for two minutes between exercise and attempts.

### 2.4. Exercise Training Program

A specialist in sports medicine and a clinical exercise physiologist supervised all training sessions. The training groups performed exercises three times a week for 12 weeks. The training program volume and intensity were designed using prescribed exercise guidelines for patients with T2D [28]. Each training session included a 5- to 10-min warm-up, 45 min of resistance training at 60–70% of 1-RM which was then followed by 30 min of aerobic exercise at 60–70% of VO_2peak_ on a motorized treadmill (Pulsar 3p, H/P/Cosmos, Nussdorf-Traunstein, Germany). Resistance exercise consisted of eight exercises: upper and lower body (leg press, knee flexion, knee extension, chest press, lat pull-down, shoulder press, abdominal crunches, biceps curls, triceps press down). In order to meet the overloading principle, the 1-RM and VO_2peak_ test were repeated every four weeks. The aerobic exercise time was 10 min during the initial weeks and was gradually increased to 30 min by the eighth week, which continued until the end of the study. The details of both the aerobic and resistance exercise training are presented in Table 1.

### 2.5. Nutrient Intake and Dietary Analysis

Participants were instructed to document their food intake as precisely as possible for three days (two weekdays and one day on the weekend) throughout the study. Total energy (kcals per day), and intakes of carbohydrates, fats and proteins (grams per day) were calculated. All dietary logs of all the subjects were assessed using Diet Analysis Plus, version 10 (Cengage, Boston, MA, USA) (Table 2). 

### 2.6. Supplementation Protocol

The broccoli sprouts supplement was purchased as a powder from Cyvex Nutrition Company (Irvine, CA, USA). The sulforaphane content of the broccoli supplements (BroccoPhane) was ~22.5 mmol/g, as determined by the manufacturer using a high-performance liquid chromatography method. The dose of sulforaphane used in this study was 225 µmol per 10 g/d of broccoli sprouts powder supplement. Both participants and researchers were blinded to supplement allocations, and a dietitian who was not in the study conducted the allocations of broccoli supplement and placebo to the groups. The two broccoli groups (TS and S) received a monthly supply of 30 packets (containing 10 g broccoli supplement) for the study duration. Participants were instructed to consume one packet of broccoli supplement powder after a daily meal (preferably with a beverage to reduce gastrointestinal complications) for 12 weeks. The two other placebo groups (TP and CP) consumed 5 g of cornstarch powder which was colored with spinach powder (ratio of 10:1) with a beverage daily. The volume, flavor, and color of the placebo were matched with the broccoli supplements. All participants were asked to maintain a regular diet and lifestyle. Moreover, participants were examined for possible side effects by a nutritionist every week during the 12 week study period. Patients who reported side effects or consumed less than 80% of the packets or changed their medications were excluded from the study.

### 2.7. Biochemical Analysis

Blood samples (10 cc) were taken from an indwelling cannula in the antecubital vein of seated subjects. Samples were collected in tubes containing EDTA (ethylenediaminetetraacetic acid) and then centrifuged at 3500 rpm for 15 min at 4 °C. Plasma concentrations of insulin were determined using a commercially available radioimmunoassay kit (Diagnostic Systems Laboratories, Webster, TX, USA), while plasma glucose concentrations were measured with a colorimetric-enzyme (glucose oxidase) method using glucose assay kits (Pars Tests Company Kit, Tehran, Iran) with a 1 mg/dL sensitivity. The HOMA-IR model was used to measure insulin resistance, where the insulin resistance index = fasting plasma glucose (mmol/L) × fasting serum insulin (μU/mL)/22.5 [29]. Plasma levels of dectin-1 were measured using an ELISA kit (RayBiotech Company, Germany) having a sensitivity of 0.082 ng/mL. Plasma triglycerides (TG), high-density lipoprotein (HDL), low-density lipoprotein (LDL), total cholesterol (TC) levels were measured with a standard biochemical analyzer (DAX 96; Bayern Diagnostics, Milan, Italy). The serum concentration of high sensitive C reactive protein (hs-CRP) was evaluated by using an enzyme-linked immunosorbent assay (ELISA) kit (Diagnostics Biochem Canada Inc., London, ON, Canada). Tumor necrosis factor-α (TNF-α) and interleukin-6 (IL-6) levels were assessed with ELISA kits (Diaclone, Besancon, France). The coefficients of variation were 1.2% for glucose, 1.8% for insulin, 2.9% for HOMA-IR, 3.1.0% for dectin-1, 2.4% for HDL, LDL, TC, TG and <5%. For hs-CRP, TNF-α and IL-6.

### 2.8. Statistical Analysis

Data are presented as the mean ± standard deviation (mean ± SD). All statistical analyses were computed using IBM SPSS statistical software (Version 23.0; SPSS, Inc., Chicago, IL, USA). The normality of the data was assessed with the Shapiro–Wilk test. The Leven test showed that the homogeneity of variances was present (*p* > 0.05). Baseline data were compared between the four groups using one-way ANOVA tests and Tukey’s post hoc tests. A repeated measures test of ANOVA (group × time) was used to compare differences in biochemical, body composition markers and dietary variables (total energy in kcal per day, intake of carbohydrates, fats, and proteins) between the four groups. If an ANOVA test determined significant differences, pairwise comparisons (planned comparisons) were used to determine which mean differences were statistically significant. Statistical significance was established at *p* ≤ 0.05. Effect size (ES) was assessed by partial eta-squared $\left(\begin{array}{c} p\\ 2 \end{array}\right)$ and repeated measures ANOVA was used to determine statistical power (1 − β). The sample size was designed to detect a difference in study variables with a 95% confidence interval (CI) and ≥80% power value.

## 3. Results

In all study groups (CP, S, TP, and TS), no significant differences (*p* > 0.05) were observed for age, body mass, BMI, FFM, fat, duration of diabetes, blood pressure (i.e., hypertension), and antidiabetic medications (Table 3). Moreover, there were no significant differences (*p* > 0.05) between the groups for study clinical and biochemical variables at baseline (Table 3).

### 3.1. Dietary Analysis

There were no differences between groups in total energy consumption and energy derived from carbohydrate, fat and protein at baseline and after 12 weeks (*p* > 0.05) (Table 2).

### 3.2. Dectin-1, CRP, IL-6 and TNF-α

**Figure 3:**
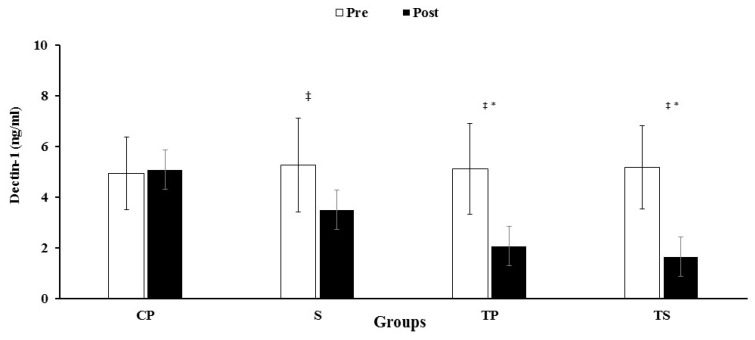
Pre and post training values (mean ± SD) for 3 Dectin-1. CP, Control–Placebo; S, Supplement; TP, Training–Placebo; TS, Training–Supplement groups. * indicates significant differences compared to the control group (*p* < 0.05). ‡ indicated significant difference from baseline (*p* < 0.05).

A significant interaction (group X time) was observed for dectin-1 (F3, 40 = 13.6, *p* = 0.0001, ES = 0.50, power = 1.0), hs-CRP (F3, 40 = 25.1, *p* = 0.0001, ES = 0.65, power = 1.0), IL-6 (F3, 40 = 42.1, *p* = 0.0001, ES = 0.76, power = 1.0), and TNF-α (F3, 40 = 40.3, *p* = 0.0001, ES = 0.75, power = 1.0). Plasma levels of dectin-1 were decreased in the S, TS and TP groups compared to the CP group (*p* < 0.05) (Figure 3). However, the decrease in the S group was not significant (*p* > 0.05) when compared to the CP group. The reduction in hs-CRP concentrations in TS, TP and S groups were not significant (*p* > 0.05) compared to the CP group (Table 3). Levels of IL-6 decreased (*p* < 0.05) in both TS, and TP groups when compared to the CP group (Table 3). However, there were no changes (*p* > 0.05) in the S group compared to the CP group (Table 3). TNF-α levels were reduced (*p* < 0.05) in the TS group when compared to the CP group, but these reductions in TP and S groups were not significant (*p* > 0.05) when compared with the CP group (Table 3). Paired t-tests showed decreases (*p* < 0.05) for hs-CRP, IL-6 and TNF-α levels after 12 weeks (compared to baseline) in the TS, TP and S groups (Table 3).

### 3.3. Insulin, HOMA-IR and Glucose

**Figure 4:**
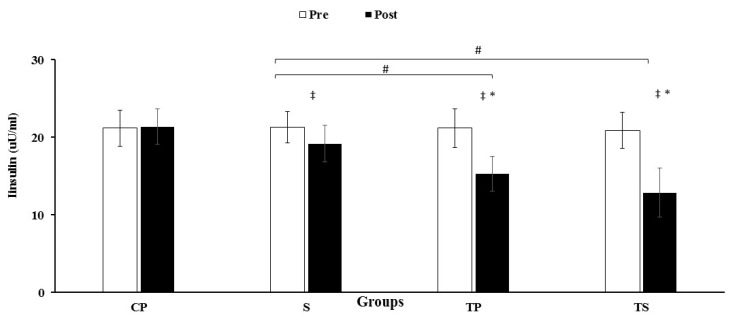
Pre and post training values (mean ± SD) for insulin in CP, Control–Placebo; S, Supplement; TP, Training–Placebo; TS, Training–Supplement groups. * indicates significant differences with control group (*p* < 0.05). * indicates significant differences compared to the control group (*p* < 0.05). # indicated significant differences compared to S group (*p* < 0.05). ‡ indicated significant difference from baseline (*p* < 0.05).

**Figure 5:**
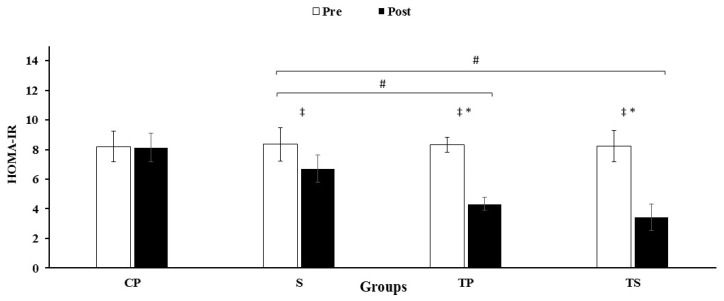
Pre and post training values (mean ± SD) for HOMA-IR in CP, Control–Placebo; S: Supplement; TP, Training–Placebo; TS, Training–Supplement groups. * indicates significant differences with control group (*p* < 0.05). * indicates significant differences compared to the control group (*p* < 0.05). # indicated significant differences compared to S group (*p* < 0.05). ‡ indicated significant difference from baseline (*p* < 0.05).

An interaction was observed between group and time for insulin (F3, 40 = 73.5, *p* = 0.0001, ES = 0.85, power = 1.0) HOMA-IR (F3, 40 = 98.7, *p* = 0.0001, ES = 0.88, power = 1.0) and glucose (F3, 40 = 52.5, *p* = 0.0001, ES = 0.79, power = 1.0). There were reductions (*p* < 0.05) in the levels of insulin, HOMA-IR and glucose in the TS, TP groups compared to the S and CP group (Figure 4 and Figure 5, Table 3). Nevertheless, these decreases were not significant (*p* > 0.05) in the S group when compared with the CP group (Figure 4 and Figure 5, Table 3). Levels of dectin-1, insulin, HOMA-IR, and glucose were decreased (*p* < 0.05) after 12 weeks when compared to baseline values in the TS, TP and S groups.

### 3.4. Lipid Profiles

There was an interaction between group and time for HDL (F3, 40 = 44.6, *p* = 0.0001, ES = 0.77, power = 1.0), LDL (F3, 40 = 82.5, *p* = 0.0001, ES = 0.86, power = 1.0), TC (F3, 40 = 325.3, *p* = 0.0001, ES = 0.96, power = 1.0) and TG (F3, 40 = 228.3, *p* = 0.0001, ES = 0.94, power = 1.0). Plasma levels of HDL were increased in the TS, TP and S groups compared with the CP group (*p* < 0.05). However, this increase was greater in the TS and TP groups compared with the S group (*p* < 0.05) (Table 3). Plasma levels of LDL, TC and TG were decreased after 12 weeks in both the TS and TP groups compared to the CP group (*p* < 0.05) (Table 3). Nevertheless, these decreases for LDL and TG were greater (*p* < 0.05) in the TS group compared to the S group. No changes were observed for LDL, TC and TG in the S group when compared to the CP group (*p* > 0.05) (Table 3). A paired sample *t*-test indicated an increase (*p* < 0.05) in HDL and also decreases (*p* < 0.05) for LDL, TC and TG from baseline to post measurements after 12 weeks in the TS, TP and S groups (Table 3).

### 3.5. Body Composition Variables and VO_2peak_

There was an interaction between group and time for weight (F3, 40 = 84.0, *p* = 0.0001, ES: 0.86, power:1.0), % body fat (F3, 40 = 40.5, *p* = 0.0001, ES: 0.75, power:1.0), BMI (F3, 40 = 62.1, *p* = 0.0001, ES: 0.82, power:1.0), FFM (F3, 40 = 22.5, *p* = 0.0001, ES: 0.62, power:1.0), and VO_2 peak_ (F3, 40 = 109.8, *p* = 0.0001, ES: 0.89, power:1.0) (*p* < 0.05). There were reductions in weight in the TS and TP groups compared to the CP group (*p* < 0.05). However, no changes in body weight were observed in the S group compared to CP group (*p* > 0.05) (Table 3). There were reductions in body fat% in both the TS and TP groups (*p* < 0.05) compared to the S and CP groups (Table 3). Body fat percent changes in the S group in comparison with the CP group were not significant (*p* > 0.05) after 12 weeks. Only the TS group had a reduction (*p* < 0.05) in BMI when compared to the CP group (Table 3). The decreases in the TP and S groups were not significant (*p* > 0.05) when compared to CP group (Table 3). Fat free mass in the both the TS and TP groups were increased (*p* < 0.05) compared with the CP group after 12 weeks of training (Table 3), while there were no changes in the S group (*p* > 0.05) compared to the CP group after 12 weeks (Table 3). Values for VO_2peak_ were increased after 12 weeks in the TS and TP groups compared with the CP and S groups (*p* < 0.05) (Table 3). There were significant changes in weight, body fat%, BMI, FFM, and VO_2peak_ from baseline to 12 weeks in the TS and TP groups (*p* < 0.05) (Table 3).

## 4. Discussion

This study indicates that plasma levels of dectin-1, HOMA-IR, insulin, and selected adipokines such as TNF-α and IL-6 as well as hs-CRP were improved after 12 weeks in the S, TS and TP groups compared to their base levels, with greater improvements in the TS group. In addition, improvements in the lipid profile, insulin levels, body composition variables, and VO_2peak_ were observed only in the TS and TP groups.

We report that dectin-1 was decreased in the TS, TP, and S groups, but that there were greater improvements in the TS (−70%) and TP (−60%) groups compared to their baseline levels. A study by Ruffino et al. (2016) indicated that eight-weeks of a moderate-intensity exercise program (walking for 45 min, 3 d/w) increased dectin-1 levels and other monocyte M2 markers in overweight females [30]. In addition, not only did we find a significant decrease in dectin-1 levels in the supplement group, but also that the improvement in the TS group was greater (but non-significant) than in the TP group, suggesting additional benefits of broccoli supplement + exercise training in altering dectin-1 levels.

Activation of dectin-1 induces the production of pro-inflammatory cytokines, chemokines [6], and reactive oxygen species [7]. We measured reductions in adipokines such as TNF-α and IL-6 as well as hs-CRP after 12 weeks in the S, TP and, TS groups when compared to their baseline levels, with non-significant decreases in the TS group compared to S and TP groups. These results support other studies reporting beneficial effects of CARET on inflammatory markers in individuals with metabolic syndrome disorders [31,32]. For example, it is shown that 16-weeks of CARET (aerobic intensity: 40% to 65% of heart rate reserve, resistance intensity: 40% to 60% of 1-RM, three times/week) reduced TNF-α and IL-6 levels in individuals with T2D [21]. The potential explanation for this alteration is that changes in IL-6 produced by exercising muscles could elevate anti-inflammatory cytokines such as IL-10 and interleukin-1 receptor antagonist (IL-1RA), which are able to decrease TNF-α levels to a significant level [21]. A recent study reported that ingestion of sulforaphane (50 mg/kg body weight) decreased the expression of some adipokines, e.g., IL-6 and TNF-α and blood biomarkers of tissue damage or cell death (alanine aminotransferase, aspartate aminotransferase, and lactate dehydrogenase) following exercise to exhaustion in male mice [33]. In addition, 10 weeks of consuming broccoli sprouts (30 g per day) improved inflammatory markers in healthy but overweight individuals [34]. Our study suggests that not only can broccoli supplement alone improve pro-inflammatory factors compared to their baseline levels, but also broccoli supplement + CARET may have additional beneficial (but non-significant) effects on improving pro-inflammatory factors such as dectin-1. The potential explanation for this improvement could be related to the finding that the consumption of sulforaphane suppresses NLRP3 (which is predominantly expressed in macrophages and triggers immune responses), and which then decreases dectin-1 levels and other pro-inflammatory factors in individuals with T2D [35]. Taken together, consuming broccoli and doing CARET can have synergistic beneficial effects on pro-inflammatory factors. In line with this, we found greater improvements in TNF-α (−48%) and IL-6 (−49%) as well as dectin-1 (−70%) levels in the TS group compared to their baseline levels. However, we did not find significant differences in pro-inflammatory factors between the S and CP groups; therefore, additional experiments are needed to better define the underlying mechanisms of improvements in inflammatory factors, particularly dectin-1, following consumption of broccoli supplement.

Our study also shows that insulin and insulin resistance (HOMA-IR) levels decreased in the S, TP, and TS groups, compared to their baseline levels with the greatest improvement in the TS group. CARET is a more potent exercise modality in improving insulin and glucose uptake in individuals with T2D [36,37,38], likely because of the different mechanisms of CARET [17,36,38]. For example, the activation of larger groups of muscle mass compared to resistance training or aerobic training alone, leading to greater improvements in insulin resistance and glucose uptake [17,38]. Additionally, improvement in insulin and glucose could be partly due to increased muscle mass and GLUT-4 induced by CARET [39]. We also measured improvements in insulin and HOMA-IR in the S and TS groups when compared with their baseline levels, with the improvements caused by broccoli consumption similar to findings in other studies [10,12]. Recent findings have suggested that IL-6 gene expression is mildly elevated in individuals with insulin resistance [40]. Furthermore, the C-174C genotype of the IL-6 gene is associated with insulin resistance in normoglycemic individuals [41]. In addition, Insulin signaling and secretion are reduced by decreasing TNF-α levels [42], and insulin resistance is directly related to TNF-α levels [38,43]. However, both CARET and broccoli can improve insulin resistance through improving IL-6 levels, decreased levels of pro-inflammatory cytokines, and reduction in body fat percentage, stimulates an anabolic and anti-inflammatory pathway that preserves lean body mass and improves insulin resistance in T2D. The largest improvements in insulin and HOMA-IR were in the TS group. One of the main reasons is that strengthening antioxidant defenses is important in inhibiting the activation of signaling pathways such as nuclear factor kappa B and extracellular signal-related kinases that cause insulin resistance [44]. Sulforaphane is an important bioactive component of broccoli supplement, which leads to lipid peroxidation and increases total antioxidant capacity [10]. Sulforaphane activates the transcription Nrf-2 (NF-E2-related factor 2) to regulate cellular redox homoeostasis by increasing the expression of enzymes that detoxify reactive oxygen species [45,46]. Thus, enhancing the endogenous antioxidant network with broccoli supplementation can suppress stress-sensitive signaling pathways and improve glucose uptake, insulin, and HOMA-IR and other long-term complications in individuals with T2D [10].

Our study also reports improvements in the lipid profiles in the S, TP, and TS groups compared with their baseline levels; however, our study failed to improve lipid profiles, except for HDL-C, in the S group when compared to the CP group. Broccoli supplementation decreases plasma LDL-C via regulation of the redox status of cells and tissues, leading to enhanced the activity of AMP-activated protein kinase (AMPK) signaling and suppression of Phosphoinositide 3-kinases (PI3K) signaling [47]. Additionally, aerobic exercise combined with weight loss reduces blood cholesterol, LDL-C, and triglycerides and also HDL-C [48]. Improvements in lipid profiles occur during weight loss in individuals with overweight/obesity [49,50]. A study by Varady et al. (2011) reported decreases in LDL levels following a calorie restriction diet, with no reductions in LDL levels after 12 weeks of moderate-intensity exercise training (three days per week, 45 min, 60% HRmax) [50]. We report greater weight loss (TS—12%, TP—7%), with our exercise program causing a higher stimulation and greater improvements in lipid profiles.

Although we tried to control energy expenditure in all the groups, body composition variables did not differ in the CP and S groups. However, body composition variables (BMI, fat% and fat free mass) were improved in the TP and TS groups, likely because CARET is a potent strategy for reducing body weight and fat mass percentage, as this exercise modality requires more energy expenditure and a higher rate of excess post-exercise oxygen consumption [17]. Additionally, a study by Lopez-Chillon et al. (2019) reported that 10 weeks of consuming broccoli sprouts (30 g/day) altered body fat levels in individuals with obesity, possibly because broccoli supplement induces an alteration in indole-3-carbinol, which decreases adipogenesis by suppressing pathways of lipid accumulation mediated by PPARγ [34]. However, we did not find an improvement in body composition variables in the S group that might stem from the dosage of supplement and/or the lack of energy intake control in our study.

Our study has several limitations. Firstly, we did not identify the mechanisms by which bioactive components of broccoli can improve dectin-1 levels. Second, our study cannot be generalized as females were not included in patient recruitment. In addition, we did not control dietary and energy expenditure, which could affect many of the physiologic variables that can modulate adipokines release. Finally, our study did not include a more heterogeneous group of individuals diagnosed with T2D.

## 5. Conclusions

This study reports that the inclusion of broccoli supplementation to a CARET program for individuals with T2D leads to greater improvements in dectin-1 levels and body composition, cardiorespiratory fitness, lipid profiles, glycemic control, and insulin resistance compared to their baseline levels. Our findings suggest that a combination of broccoli supplement with CARET has additive beneficial effects in individuals with T2D. An important finding of our study is that supplementing with broccoli and CARET causes greater elevations in VO_2peak_ in patients with T2D.

## Figures and Tables

**Table 1:** CARET (Combine Aerobic + resistance Exercise Training) for 12 weeks.

Weeks	Aerobic Training			Resistance Training			
	Duration (min/Sessions)	Intensity (VO_2peak_)	Sessions Per Week	Sets	Repetitions	1-RM	Sessions Per Week
**1 to 2**	10	60%	3	3	12–15	60–65%	3
**2 to 4**	15	65%	3	3	12–15	60–65%	3
**4 to 6**	20	70%	3	3	8–10	70%	3
**6 to 8**	25	70%	3	3	8–10	70%	3
**8 to 12**	30	70%	3	3	8–10	70%	3

1-RM, one-repetition maximum.

**Table 2:** Mean ± SD values of nutritional intake in the four study groups.

	CP		S		TP		TS	
	Pre	Post	Pre	Post	Pre	Post	Pre	Post
**Energy (kcal/d)**	2364.5 ± 164.6	2383.1 ± 106.6	2370.1 ± 162.9	2385.6 ± 157.0	2362.1 ± 214.2	2340.3 ± 213.4	2393.6 ± 215.5	2358.0 ± 193.2
**CHO (g/d)**	295.1 ± 24.0	305.1 ± 36.1	298.0 ± 33.1	304.6 ± 34.8	289.0 ±31.5	291.6 ± 29.5	292.7 ± 31.9	295.0 ± 32.4
**Fat (g/d)**	79.8 ± 16.8	77.0 ± 15.7	79.6 ± 15.8	76.9 ± 14.2	81.4± 16.0	78.9 ± 14.5	83.8 ± 17.0	79.1 ± 14.6
**Protein (g/d)**	116.3 ± 16.9	117.3 ± 17	115.3 ± 19.2	118.7 ± 19.2	118.2 ± 17.4	115.9 ± 18.7	117.0 ± 18.9	116.3 ± 17.8

CP, Control–Placebo; S, Supplement; TP, Training–Placebo; TS, Training–Supplement; CHO, carbohydrate.

**Table 3:** Mean ± SD values of glucose, lipid profile, inflammatory markers, body composition and VO_2peak_ for the four study groups.

	CP		S		TP		TS	
	Pre	Post	Pre	Post	Pre	Post	Pre	Post
**Glucose (mmol/L)**	8.71 ± 0.73	8.58 ± 0.65	8.78 ± 0.75	7.89 ± 0.75 ^‡^	8.79 ± 0.71	6.38 ± 0.47 ^‡^*^#^	8.86 ± 0.71	5.94 ± 0.54 ^‡^*^#^
**HDL-C (mmol/L)**	0.84 ± 0.05	0.84 ± 0.03	0.94 ± 0.04	0.99 ± 0.04 ^‡^*	0.92 ± 0.06	1.16 ± 0.09 ^‡^*^#^	0.90 ± 0.06	1.16 ± 0.06 ^‡^*^#^
**LDL (mmol/L)**	4.11 ± 0.12	4.08 ± 0.12	4.06 ± 0.13	3.98 ± 0.20 ^‡^	4.11 ± 0.12	3.74 ± 0.13 ^‡^*	4.20 ± 0.12	3.46 ± 0.21 ^‡^*^#^
**TC (mmol/L)**	6.20 ± 0.25	6.23 ± 0.26	6.07 ± 0.26	5.99 ± 0.28 ^‡^	6.05 ± 0.24	5.43 ± 0.23 ^‡^*^#^	6.12 ± 0.28	5.32 ± 0.23 ^‡^*^#^
**TG (mmol/L)**	6.20 ± 0.023	6.20 ± 0.28	2.23 ± 0.28	6.10 ± 0.28 ^‡^	6.12 ± 0.29	5.64 ± 0.36 ^‡^*	6.23 ± 0.29	5.43 ± 0.34 ^‡^*^#^
**hs-CRP (ng/mL)**	4.20 ± 1.02	4.25 ± 1.04	4.51 ± 1.84	3.75 ± 1.62 ^‡^	4.53 ± 1.16	3.05 ± 1.01 ^‡^	4.22 ± 1.21	2.48 ± 0.56 ^‡^
**IL-6 (pg/mL)**	4.89 ± 0.90	4.96 ± 1.09	4.90 ± 1.59	4.59 ± 1.34 ^‡^	4.98 ± 1.05	2.85 ± 1.01 ^‡^*	5.22 ± 1.21	2.64 ± 2.01 ^‡^*
**TNF-α (pg/mL)**	14.60 ± 3.18	14.78 ± 3.29	14.97 ± 4.32	13.45 ± 3.89 ^‡^	14.55 ± 4.36	11.53 ± 3.56 ^‡^	14.97 ± 3.80	7.78 ± 2.63 ^‡^*
**Weight (kg)**	86.36 ± 4.56	86.72 ± 3.90	85.00 ± 3.31	85.18 ± 3.57	86.09 ± 4.41	79.90 ± 3.53 ^‡^*	86.63 ± 4.90	79.09 ± 3.78 ^‡^*
**FFM (kg)**	28.36 ± 1.56	28.27 ± 1.84	29.63 ± 2.06	29.45 ± 2.29	28.90 ± 1.44	32.27 ± 2.05 ^‡^*	28.72 ± 1.73	32.09 ± 2.30 ^‡^*
**Fat (%)**	29.54 ± 1.12	29.63 ± 2.50	29.81 ± 2.04	29.45 ± 1.91	29.54 ± 1.75	25.90 ± 1.81 ^‡^*^#^	30.18 ± 1.83	25.09 ± 2.21 ^‡^*^#^
**BMI (kg/m^2^)**	28.34 ± 1.13	28.46 ± 0.96	27.38 ± 1.34	27.43 ± 1.33	28.51 ± 0.84	27.29 ± 0.74 ^‡^	28.06 ± 0.82	26.17 ± 0.78 ^‡^*
**VO_2peak_ (mL/kg/min)**	28.09 ± 1.97	27.63 ± 2.29	28.27 ± 2.45	28.36 ± 2.33	28.81 ± 3.45	32.90 ± 3.91 ^‡^*^#^	28.09 ± 2.42	33.36± 0.69 ^‡^*^#^

CP, Control–Placebo; S, Supplement; TP, Training–Placebo; TS, Training–Supplement; HDL, high-density lipoprotein; LDL, low-density lipoprotein; TC, total cholesterol; TG, triglyceride; hs-CRP, high-sensitivity C-reactive protein; IL-6, interleukin-6; TNF-α, tumor necrosis factor-α. FFM, fat free mass; FM, fat mass; BMI, body mass index. * indicates significant differences compared to the control group (*p* < 0.05). ^#^ indicates significant differences compared to S group (*p* < 0.05). ^‡^ indicated significant difference from baseline (*p* < 0.05).

## Data Availability

The datasets generated for this study are available on request to the corresponding author.
